# Selection and validation of reference genes for quantitative RT-PCR expression studies of the non-model crop *Musa*

**DOI:** 10.1007/s11032-012-9711-1

**Published:** 2012-06-08

**Authors:** Nancy Podevin, An Krauss, Isabelle Henry, Rony Swennen, Serge Remy

**Affiliations:** 1 Laboratory of Tropical Crop Improvement, Department of Biosystems, Faculty of Bioscience Engineering, K.U. Leuven, Kasteelpark Arenberg 13, 3001 Leuven, Belgium; 2 Present Address: European Food Safety Authority (EFSA), Largo N. Palli 5/A, 43121 Parma, Italy; 3 Present Address: Roche Diagnostics Belgium, Schaarbeeklei 198, 1800 Vilvoorde, Belgium; 4 Present Address: Section of Plant Biology and Genome Center, UC Davis, 451 E, Health Sciences Drive, Davis, CA 95616 USA; 5 Bioversity International, K.U. Leuven, Kasteelpark Arenberg 13, 3001 Leuven, Belgium

**Keywords:** *Musa*, Banana, Reference genes, qPCR, Gene expression, *Mycosphaerella*

## Abstract

Gene expression analysis by reverse transcriptase real-time or quantitative polymerase chain reaction (RT-qPCR) is becoming widely used for non-model plant species. Given the high sensitivity of this method, normalization using multiple housekeeping or reference genes is critical, and careful selection of these reference genes is one of the most important steps to obtain reliable results. In this study, reference genes commonly used for other plant species were investigated to identify genes displaying highly uniform expression patterns in different varieties, tissues, developmental stages, fungal infection, and osmotic stress conditions for the non-model crop *Musa* (banana and plantains). The expression stability of six candidate reference genes was tested on six different sample sets, and the results were analyzed using the publicly available algorithms geNorm and NormFinder. Our results show that variety, plant material, primer set, and gene identity can all influence the robustness and outcome of RT-qPCR analysis. In the case of *Musa*, a combination of three reference genes (*EF1*, *TUB* and *ACT*) can be used for normalization of gene expression data from greenhouse leaf samples. In the case of shoot meristem cultures, numerous combinations can be used because the investigated reference genes exhibited limited variability. In contrast, variability in expression of the reference genes was much larger among leaf samples from plants grown in vitro, for which the best combination of reference genes (*L2* and *ACT* genes) is still suboptimal. Overall, our data confirm that the stability of candidate reference genes should be thoroughly investigated for each experimental condition under investigation.

**Electronic supplementary material:**

The online version of this article (doi:10.1007/s11032-012-9711-1) contains supplementary material, which is available to authorized users.

## Introduction

Reverse transcription quantitative polymerase chain reaction (RT-qPCR) is a routinely used technique for gene expression analysis because of its main advantages of relatively low cost, good speed, a wide dynamic range, and feasibility in non-model organisms (Thellin et al. 1999). However, extreme care needs to be exercised in the interpretation of RT-qPCR data and, in particular, normalization is crucial to control for experimental errors that can be introduced at a number of stages throughout the procedure (reviewed in Bustin 2002; Deepak et al. 2007; Gachon et al. 2004; Guenin et al. 2009; Huggett et al. 2005; Nolan et al. 2006; Radonic et al. 2004). The most reliable method of normalization involves the use of one or preferably more housekeeping or reference genes as internal standards. The expression of these reference genes is therefore expected to remain constant under different experimental conditions. Commonly used reference genes are cellular maintenance genes, which regulate basic and ubiquitous cellular functions such as components of the cytoskeleton, glycolytic pathway, protein folding, synthesis of ribosome subunits, electron transport, and protein degradation (Gachon et al. 2004; Huggett et al. 2005). Recent studies have shown that the transcriptional levels of these reference genes are not always stable, and that no single reference gene has a constant expression level under all experimental conditions (Dheda et al. 2005; Gutierrez et al. 2008a; Schmittgen et al. 2000; Thellin et al. 1999; Tricarico et al. 2002; Vandesompele et al. 2002). However, according to a recent metastudy, many of the published articles on plant gene expression still rely solely on one reference gene for normalization (Gutierrez et al. 2008b). Different statistical procedures or software packages have been reported to identify the best suitable reference gene(s) for a sample set, such as geNorm (Vandesompele et al. 2002), NormFinder (Andersen et al. 2004), ΔCt approach (Livak and Schmittgen 2001), Bestkeeper (Pfaffl et al. 2004), and “Stability index” (Brunner et al. 2004). For plants, multiple reference genes have been analyzed in the model plants *Arabidopsis thaliana* (Czechowski et al. 2005; Graeber et al. 2011; Hong et al. 2010; Lilly et al. 2011; Remans et al. 2008; Rieu et al. 2008), tobacco (Schmidt and Delaney 2010), and rice (Jain et al. 2006; Kim et al. 2003). Recently, studies have also been published on vegetables (Castro et al. 2011; Die et al. 2010; Expósito-Rodriguez et al. 2008; Garg et al. 2010; Gutierrez et al. 2011; Hu et al. 2009; Libault et al. 2008; Mascia et al. 2010; Migocka and Papierniak 2011; Nicot et al. 2005; Obrero et al. 2011; Wan et al. 2010), fruits (Reid et al. 2006; Tong et al. 2009), cereals and grasses (Dombrowski and Martin 2009; Hong et al. 2008; Jarosova and Kundu 2010; Lee et al. 2010; Paolacci et al. 2009; Silveira et al. 2009), trees (Brunner et al. 2004; Li et al. 2011; Goncalves et al. 2005), and a variety of other plant species (Artico et al. 2010; Cordoba et al. 2011; Cruz et al. 2009; Dong et al. 2011; Iskandar et al. 2004; Mallona et al. 2010; Maroufi et al. 2010; Tu et al. 2007; Yang et al. 2010). While this article was being reviewed, Chen et al. (2011) published the first report describing the validation of reference genes in dessert banana that mainly focuses on fruit tissues.

*Musa* (bananas and plantains, collectively referred to as banana) species provide a staple food in many developing countries and with an annual production of more than 130 million tons per year it is the fourth most important food crop worldwide (FAO 2009). Diseases and pests (Jones 2009) as well as abiotic stresses including drought and temperature changes (Israeli and Lahav 2000; van Asten et al. 2011) are amongst the major and increasingly damaging constraints on banana production. Our aim is to provide tools for investigating the expression of genes involved in stress responses of non-fruit tissues of banana, with the ultimate goal of gaining further insight in the molecular mechanisms underlying the interactions between banana plants and their environment.

Banana is a typical non-model crop with limited genomic and cDNA/expressed sequence tag (EST) sequences available. The Global *Musa* Genome Consortium (GMGC) (Global Musa Genomics Consortium 2011) reports that currently less than 1 % of the *Musa* genome is sequenced (Carpentier et al. 2008). Therefore, as for most non-model crops, the possibilities for gene expression analyses in banana species are limited. For example, no microarray slides are available and the lack of a reference sequence makes next-generation RNA-Seq analyses difficult. There is a need for alternative techniques such as SuperSAGE (Coemans et al. 2005) and RT-qPCR. For studies on banana, an actin, a 25S ribosomal protein, a pectate lyase and *GAPDH* have been used as unique reference genes for expression experiments (Elitzur et al. 2010; Mbeguie-Mbeguie et al. 2007; Shekhawat et al. 2011; Thomas-Hall et al. 2007; van den Berg et al. 2007; Wang et al. 2010). In this study, we validated candidate reference genes for expression studies in banana plants by evaluating their robustness under different conditions, and in different tissues and varieties.

## Materials and methods

### Plant material and growth conditions

A summary of the different types of cultures, tissues, and varieties used is provided in Table 1.

**Table 1:** Summary of the experiments, varieties, cultures/tissues, and experimental treatments

Experiment	Variety (genomic group)	Culture type/tissue	Experimental treatment
In vitro	Grand Nain (AAA)	In vitro plants/pooled leaves	Effect of acetone
GH^a^ development	Tuu Gia (AA)	Greenhouse plants/leaf	Gene expression at different time points
GH^a^ varieties	Tuu Gia (AA)	Greenhouse plants/leaf	Variation in gene expression among varieties
	Yangambi Km5 (AA)		
Leaf disc	Tuu Gia (AA)	Leaf discs	Effect of *Mycosphaerella fijiensis* inoculation
Meristem sucrose	Cachaco (ABB)	In vitro meristem cultures	Effect of sucrose-induced osmotic stress
Meristem varieties	Cachaco (ABB)	In vitro meristem cultures	Variation in gene expression among varieties
	Mbwazirume (AAAh^b^)		
	Williams (AAA)		

^a^ *GH* greenhouse

^b^Highland banana

#### In vitro plantlets

Plants of the variety Grand Nain [AAA Cavendish sub-group; International Transit Centre (ITC accession number 0180)], were grown on semi-solid regeneration medium [REG: MS medium supplemented with vitamins (Murashige and Skoog 1962), 1 μM benzyladenine, 1 μM indole acetic acid, 10 mg l^–1^ ascorbic acid, 0.09 M sucrose, and 3 g l^−1^ Gelrite^®^] at 26 ± 2 °C under a 16-h photoperiod with a photosynthetic photon flux density of 50 μE m^−2^ s^−1^ provided by Cool White fluorescent lamps (TLD 58 W/33; Philips, France). After 5.5 weeks of growth, the plants were transferred to a liquid REG medium. After 2 months, fresh liquid REG medium was added, and to half of the plants, acetone was supplemented to a final concentration of 0.5 % (v/v). Acetone treatment was tested since acetone is used to dissolve certain biologically active compounds in the author’s laboratory. Leaves were harvested 2 days after the addition of acetone from six and seven plants grown on the REG medium without and with 0.5 % (v/v) acetone, respectively.

#### Greenhouse plants

Plants of the varieties Tuu Gia (AA, ITC.0610) and Yangambi Km5 (AAA Ibota sub-group, ITC.1123) were grown in pots in the greenhouse where the photoperiod was extended to 12 h by artificial light, if required. The temperature reached 26 °C in the day and 18 °C in the night, and the relative humidity ranged between 70 and 90 %. For the “development” experiment, the first sampling was performed using the second unfolded leaf of each of the 6 Tuu Gia plants (age, 6 months; time point, Ta) and the same leaf was sampled 16 (Tb) and 26 (Tc) days later. For the “variety” experiment, the leaf tissue of Tuu Gia at the Tc stage was compared to the leaf tissue of the age-matched Yangambi Km5 (Tc).

#### Leaf disc

Leaf disc infection was done essentially as described previously (Abadie et al. 2008). Briefly, 5 × 5 cm discs of the first unfolded leaf of 5- to 6-month-old greenhouse Tuu Gia plants were excised, rinsed multiple times with sterile water, and placed with the adaxial side onto 0.4 % (w/v) agar medium containing 8 mg l^−1^ gibberellic acid. The leaf discs were sprayed with a solution containing 2 × 10^4^ *Mycosphaerella fijiensis* conidia in sterile water or with sterile water alone. The leaf discs were incubated at 26 °C under a 12-h photoperiod for 2 weeks. Eight whole leaf discs were sampled for each group 15 days after incubation of the leaf discs, i.e., at the time that the first symptoms of infection appeared in the sprayed group.

#### Meristems

Multiple shoot meristem cultures of Cachaco (ABB, cooking banana, ITC.0643), Mbwazirume (AAAh, East African highland banana, ITC.0084), and Williams (AAA Cavendish sub-group, ITC.0365) were initiated as previously described (Strosse et al. 2006) and maintained in the dark on a proliferation medium (P4; MS medium supplemented with vitamins (Murashige and Skoog 1962), 100 μM 6-benzylaminopurine, 1 μM indole acetic acid, 10 mg l^−1^ ascorbic acid, 0.09 M sucrose, and 2.5 g l^−1^ Gelrite^®^). For the “variety” experiments, meristems were harvested 6 days after subculture. For the “sucrose” experiment, Cachaco meristems were divided into three groups. Samples from all groups were subcultured on day 0 and placed back onto their growth medium. Samples from the cutting control group were harvested 24 h later. The plants in the “control” and “sucrose” groups were transferred to a fresh P4 medium (0.09 M sucrose) and P4 medium containing 0.4 M sucrose, respectively, on day 4; meristems were harvested 2 days later (day 6). Samples from five meristems were collected for each group.

### In silico identification of candidate reference genes

Candidate reference genes were identified by literature search, with emphasis on reference genes previously used in plants. As indicated in Electronic Supplementary Material 1, the genes included in this study have different cellular functions. A BlastX similarity search (Altschul et al. 1990) was performed against the *Musa* 3′ EST database (donated to the GMGC by Syngenta) as well as all publicly available sequences in GenBank. One or more primer pairs were designed for each sequence using the Primer3 program (Primer3 2011) and the following parameters: length, 19–25 bp; optimal *T*_m_, 57–61 °C; GC %, 45–60 % and amplicon length, 75–200 bp. Subsequently, primer pairs were tested for heteroduplex formation using the OligoAnalyzer 3.1 program (OligoAnalyzer 2011). Before RT-qPCR, the primer pairs were tested by gradient RT-PCR using the Mastercycler Gradient PCR machine (Eppendorf, Hamburg, Germany) to identify the optimal annealing temperature. Reactions contained 1 × ThermoPol reaction buffer [New England Biolabs (UK) Ltd., Hitchin, United Kingdom], 200 μM of each dNTP, 500 nM of reverse and forward primers, 0.0125 U μl^−1^ *Taq* DNA polymerase [New England Biolabs], 1–2 μl cDNA template, and water to reach a total volume of 20 μl. Amplification was achieved via the following program: initial denaturation at 95 °C for 3 min 30 s followed by 30 cycles of 95 °C for 20 s, 62.5 ± 6.5 °C for 30 s, and 72 °C for 20 s, with a final elongation at 72 °C for 5 min. Amplicon size was verified by 1.5 % (w/v) agarose gel electrophoresis.

### Total RNA extraction

The plant material was harvested, snap frozen in liquid nitrogen, and stored at −80 °C. Total RNA was extracted from different plant tissues using the RNeasy^®^ Plus Mini Kit or RNeasy^®^ Midi Kit (Qiagen, Hilden, Germany), according to the manufacturer’s instructions except for the addition of PVP40,000 to the lysis buffer at a final concentration of 5 mg ml^−1^. The extracted RNA was treated with RNase-free Ambion^®^ DNaseI (AB Applied Biosystems, Lennik, Belgium), which was subsequently removed during a phenol–chloroform/ethanol purification step. The quantity and quality (*A*_260/230_ and *A*_260/280_) of total RNA were determined using the Nanodrop ND-1000™ spectrophotometer (Nanodrop Technologies, Wilmington, DE, USA). Finally, to verify the absence of gDNA in the RNA samples, a qPCR was performed using DNase-treated RNA as template and primers for the *EF1* gene. The reaction mixture was identical to that of the RT-qPCR (see below, Two-step real-time RT-PCR section) except that λ-DNA was omitted and instead of 2 μl cDNA template, 1 μl RNA was used. Following the initial polymerase activation at 95 °C for 15 min, 40 cycles of 95 °C for 15 s, 60 °C for 20 s, and 72 °C for 20 s were run. Finally, a melting program as described below (see below, Two-step real-time RT-PCR section) was executed at the end of the real-time PCR run. Only samples for which no amplification could be detected, thereby indicating the absence of DNA contamination, were used.

As the efficiency of enzymes used for PCR is affected by the quality of the RNA samples (Schmittgen and Zakrajsek 2000), only RNA samples with OD_260/280_ ratios above 1.6 and OD_260/230_ ratios above 1.8 were used for further analysis. These ratios indicate minimal presence of protein contaminants and organic pollutants, respectively, and were experimentally determined because RNA samples not meeting these criteria yielded irreproducible results with relatively high Ct values (data not shown). Additionally, only RNA samples for which absence of DNA could be ascertained using a RNA qPCR test were further processed.

### Two-step real-time RT-PCR

One microgram of each DNA-free RNA sample was reverse-transcribed to cDNA by using an oligo(dT)_18_ primer and the RevertAid H Minus First Strand cDNA Synthesis Kit (Fermentas, St-Leon Rot, Germany) according to the manufacturer’s instructions. Real-time RT-PCR was performed on the Corbett Rotor-Gene 3000 (Qiagen, Hilden, Germany) using the SYBR Green I technology. In a total volume of 25 μl, the master mix containing 1 × ABsolute™ QPCR SYBR^®^ Green Mix (Thermo Scientific, Epsom, UK), 150 nM of each specific sense and anti-sense primers (Table 2), and 125 ng λ-DNA (Roche Diagnostics, Vilvoorde, Belgium) was mixed with 2 μl of a 50 × diluted template cDNA, control gDNA or water. λ-DNA was added as carrier DNA to minimize absorption and Poisson effects. The following amplification program was used: polymerase activation at 95 °C for 15 min, followed by 45–50 cycles of 95 °C for 15 s, 52–62 °C for 20 s, and 72 °C for 20 s, with a final elongation at 79–81 °C for 15 s. The fluorescence measurement was performed at a temperature of 79–81 °C. To verify the specificity of the amplicon for each primer pair, a melting curve was produced from 55 to 95 °C at the end of each RT-qPCR run. A minimum of five samples from each run were analyzed by agarose gel electrophoresis to verify that the product was a single band of the correct size. A standard curve of six serial four-fold dilutions of pooled cDNA, a no-template control, and the cDNA samples each with two technical replicates were always run concurrently in each assay. The cycle fit-point or threshold Ct was determined for each PCR reaction. When the values of the duplicated samples differed by more than 0.5 cycles, the measurements were repeated or discarded. The incidence of such a difference was rather rare (on average less than one sample per run of 72 samples) irrespective of the experiment or combination of reference genes. Real-time PCR efficiency was determined for each gene by using the slope of a linear regression model of the dilution series [*E* = 10^(−1/slope)^] (Pfaffl 2001; Rasmussen 2001) (Table 2). All PCR reactions displayed a correlation coefficient *R* ^2^ of above 0.98. The Ct values were imported into Microsoft Excel for further analysis.

**Table 2:** Selected candidate reference genes, primers, annealing temperatures, amplicon lengths, and actual amplification efficiencies

Gene	Primers	Sequence	Annealing temp. (°C)^a^	Amplicon length (bp)	E (±SD)^b^
*ACT11*	act11-F3	CCCAAGGCAAACCGAGAGAAG	60	150	1.00 (0.031)
	act11-R2	GTGGCTCACACCATCACCAG			
*ACT*	act-1	GAGAAGATACAGTGTCTGGA	52	231	0.88 (0.073)
	act-2	ATTACCATCGAAATATTAAAAG			
*EF1*	EF1-F2	CGGAGCGTGAAAGAGGAAT	62	185	0.99 (0.069)
	EF1-R2	ACCAGCTTCAAAACCACCAG			
*L2*	L2-F2	AGGGTTCATAGCCACACCAC	61	100	1.00 (0.064)
	L2-R2	CCGAACTGAGAAGCCCCTAC			
*25S*	25S-1	ACATTGTCAGGTGGGGAGTT	59	106	0.79 (0.053)
	25S-2	CCTTTTGTTCCACACGAGATT			
*TUB*	tub-F1	TGTTGCATCCTGGTACTGCT	61	112	0.98 (0.032)
	tub-R1	GGCTTTCTTGCACTGGTACAC			

^a^As determined by gradient PCR

^b^Efficiency of PCR amplification (±standard deviation)

### Analysis of the data

The Ct values were converted into relative quantities or expression levels according to the data obtained for the samples of the dilutions series, which are used to create standard curves. Next, the reference gene stability factor (*M*), defined as the average pair-wise variation between a particular reference gene and all of the other candidate reference genes, was determined using geNorm v3.4 (Vandesompele et al. 2002). Additionally, the same values used as input data for geNorm were analyzed using the NormFinder algorithm (Andersen et al. 2004). Grouping of samples for Normfinder analyses was done according to the treatments described above (with or without acetone, different developmental time points, different varieties, with or without *M. fijiensis* inoculation, different sucrose treatments, and different varieties for the in vitro, GH development, GH varieties, leaf discs, meristem sucrose and meristem varieties experiment, respectively). ANOVA was used to determine whether differences in the Ct levels between the different experimental treatments within each experiment were significant.

## Results

### Selection of candidate reference genes and primer design

Reference genes commonly used for other plant species were investigated to identify genes displaying highly uniform expression patterns in different varieties, tissues, developmental stages, and stress conditions for the non-model crop *Musa* (banana and plantains). Nine genes from different functional groups were chosen: 18S rRNA, glyceraldehyde-3-phosphate dehydrogenase (*GAPDH*), elongation factor-1α (*EF1*), polyubiquitin, actin11 (*ACT11*), α-tubulin, β-tubulin (*TUB*), cyclophilin, and ribosomal protein L2 (*L2*) genes. Banana genes and EST fragments belonging to these gene families were identified by conducting similarity searches (BlastX). The identity of the coding sequence between *Arabidopsis* or rice and *Musa* varied between 80 and 97 % (Electronic Supplementary Material 1). At the time this study was performed, no orthologous *Musa* sequences of sufficient length could be identified for *GAPDH*, 18S rRNA, and α-tubulin.

Primer pairs were designed for the *ACT11*, cyclophilin, *EF1*, *L2*, *TUB*, and polyubiquitin genes (Electronic Supplementary Material 1). Using data from other plant species, a primer pair spanning an intron was designed for *ACT11*; this was not possible for the other genes. To ensure that each primer pair resulted in the production of a single PCR product, gradient PCR was performed on genomic DNA (gDNA) and on cDNA from leaves. For *ACT11*, *EF1*, *L2*, and *TUB*, a suitable primer pair was identified (Table 2; Electronic Supplementary Material 1). Different primer pairs for the cyclophilin and polyubiquitin genes were designed, butno product and multiple bands, respectively, were observed (Electronic Supplementary Material 1). Additionally, 25S rRNA (*25S*) and actin (*ACT*) genes that have been previously used in other banana gene expression studies (Mbeguie-Mbeguie et al. 2007; van den Berg et al. 2007) were included in our analyses. Gradient PCR was performed using previously published primer sequences and the production of one PCR product was confirmed (Electronic Supplementary Material 1). Next, the optimal primer concentration was determined for each primer pair during the first RT-qPCR analysis. Primer concentrations that resulted in the lowest threshold cycles (Ct) along with minimal primer dimers were selected and corresponded to 150 nM for all primers. An overview of the selected primers for RT-qPCR and the expected amplicon sizes is given in Table 2.

### Expression analysis

The expression levels of our candidate reference genes were determined in six different experimental set-ups using a total of 78 samples (Table 1). The different types of plant materials analyzed were leaves from in vitro and greenhouse plants, leaf discs from greenhouse plants, and in vitro meristem cultures. Samples were obtained from multiple varieties and exposed to various biotic and abiotic stress conditions (Table 1). The stability of the candidate reference gene expression was examined at the transcript level by RT-qPCR and the results were analyzed using standard statistical analysis and publicly available algorithms NormFinder and geNorm. Within a single experiment, aliquots of the same cDNA synthesis reaction were used for RT-qPCR amplification of all candidate reference genes.

### Analysis of candidate reference genes in leaf tissue of in vitro plants

**Fig. 1:**
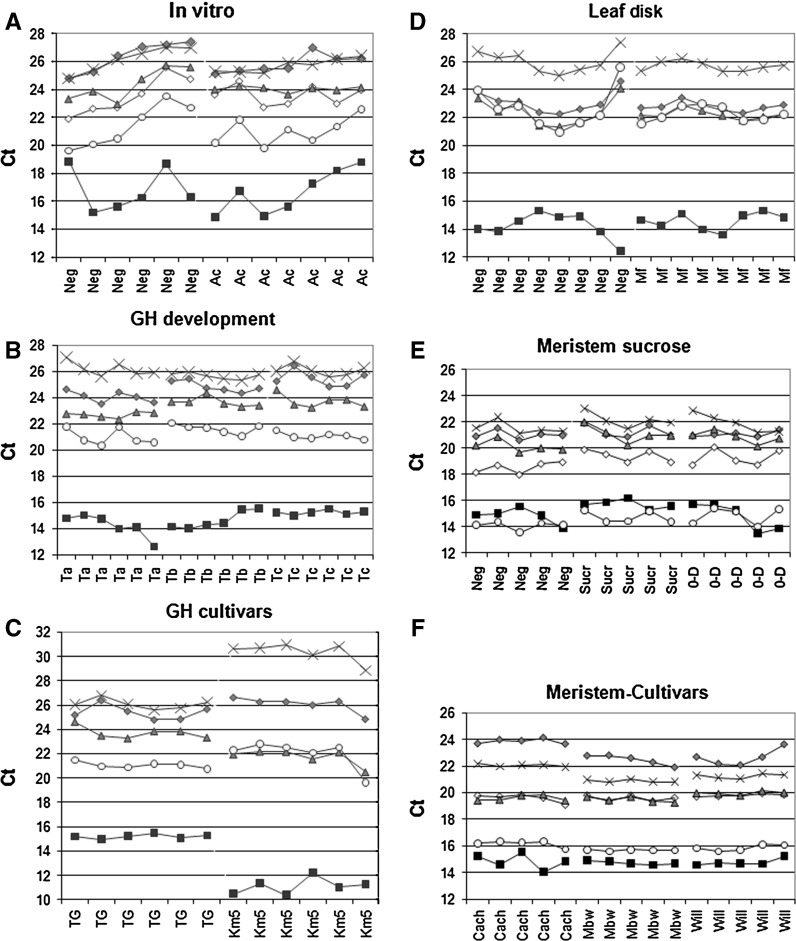
Transcriptional profiles of candidate reference genes expressed as absolute Ct values. For each sample group, about 5–8 biological replicates were analyzed. The following reference genes were tested: *25S* 25S rDNA (*filled square*), *ACT* actin (*filled diamond*), *ACT11* actin11 (*open diamond*), *TUB* β-tubulin (*filled triangle*), *L2* ribosomal protein L2 (*multiplication sign*), and *EF1* elongation factor-1α (*filled circle*). **a** Leaf tissue from in vitro cultured plants grown in a medium containing 0.5 % (v/v) acetone (*Ac*) or control medium without acetone (*Neg*). **b** Leaf tissue of greenhouse plants sampled at three different time points (*Ta* samples were harvested 6 months after transfer of the plants to the greenhouse, and *Tb* and *Tc* samples were harvested 16 and 26 days later, respectively). **c** Leaf tissue of greenhouse plants of the varieties Tuu Gia (*TG*) and Yangambi Km5 (*Km5*) at Tc. **d** Leaf discs inoculated with *M. fijiensis* conidia (*Mf*) and control leaf discs (*Neg*). **e** Meristems cut on day 0 and subsequently either placed on control medium on day 4 and harvested on day 6 (Control samples; *Neg*) or placed on high sucrose medium on day 4 and harvested on day 6 (Sucrose samples; *Sucr*) or simply harvested on day 1 (cut samples; *0*-*D*). **f** Meristems from the varieties Cachaco (Cach), Mbwazirume (Mbw), and Williams (Will) all harvested 6 days after the last cutting and subcultured on the control medium

The leaves of each of the six and seven plants grown on the REG medium without and with 0.5 % (v/v) acetone, respectively, were pooled and used for RNA isolation and cDNA synthesis. The Ct values of the six different candidate reference genes exhibited broad variability between the samples, irrespective of the acetone treatment (from 2.3 for *L2* up to 4.0 for *25S*; Fig. 1a). Firstly, within-group variation was analyzed using ANOVA and showed that the Ct values of the reference genes were not significantly different between the samples obtained from plants grown on the medium with acetone and those grown without acetone (ANOVA, *p* > 0.05).

Secondly, NormFinder was used to determine the stability of the different reference genes. This software ranks candidate reference genes according to their expression stability in an experiment (Andersen et al. 2004). NormFinder can consider the different treatment/sample groups by using a grouping function. The ranking obtained by NormFinder analysis with or without the grouping function as summarized in Table 3 was *25S*—*EF1*—*ACT11*—*ACT*—*TUB*—*L2* from least to most stable reference gene, with the best combination being *TUB* and *L2*.

**Table 3:** Stability of candidate reference genes calculated by NormFinder for the six experiments analyzed

	In vitro^a^		GH development^b^		GH varieties^c^		Leaf discs		Meristem sucrose		Meristem varieties	
Grouping	Yes	No	Yes	No	Yes	No	Yes	No	Yes	No	Yes	No
*25S*	0.353	0.861	0.275	0.401	1.453	1.606	0.290	0.902	0.229	0.469	0.180	0.224
*EF1*	0.176	0.433	0.278	0.306	0.458	0.228	0.177	0.513	0.150	0.269	**0.056**	**0.096**
*TUB*	0.112	0.293	0.280	0.339	0.811	0.739	0.022	0.092	**0.125**	**0.118**	0.193	0.216
*L2*	**0.097**	**0.239**	0.327	0.373	1.530	1.840	0.024	0.065	0.141	0.276	0.205	0.235
*ACT*	0.136	0.339	**0.242**	**0.289**	**0.312**	**0.228**	**0.019**	**0.065**	0.163	0.228	0.307	0.339
*ACT11*	0.145	0.398	ND	ND	ND	ND	ND	ND	0.148	0.288	0.207	0.201

^a^Leaf tissue

^b^Greenhouse development, leaf tissue

^c^Greenhouse varieties, leaf tissue

*Bold* the most stable gene; *underlined* the best reference gene pair. *ND* not determined

**Fig. 2:**
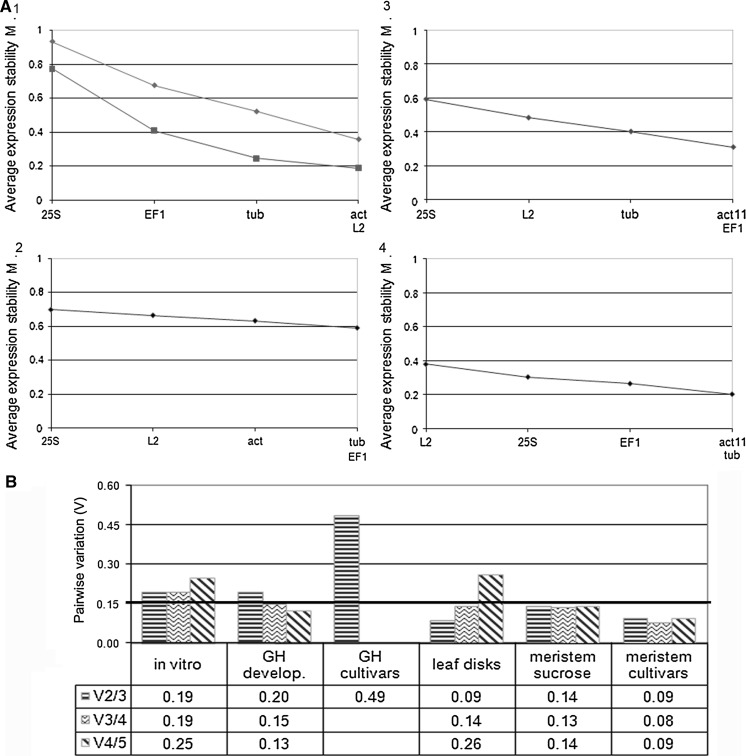
Expression stability and variation analyses of the candidate reference genes by geNorm. a Average expression stability (M) and ranking of the candidate reference genes. The lower average expression stability M indicates a more stable expression. Experiments: (*1*) In vitro (*filled diamond*), leaf discs (*filled square*), (*2*) Greenhouse development, (*3*) meristem sucrose, (*4*) meristem varieties. **b** Pair-wise variation (V) analysis of the candidate reference genes. This analysis was conducted to determine the optimal number of reference genes required for normalization. Six experimental set-ups were included in the analysis: in vitro, greenhouse (*GH*) development, *GH* varieties, leaf discs, meristem sucrose, and meristem varieties. A cut-off V-value of 0.15, below which the inclusion of additional reference genes is not required, has been proposed by Vandesompele et al. (2002) and is indicated by a *bold line*. For the greenhouse development experiment, two reference genes had an M value above 1.5 and only three genes were used to calculate the V pair-wise variation and therefore V3/4 and V4/5 could not be calculated. Abbreviations: *ACT11*: actin11; *ACT*: actin; *EF1*: elongation factor-1α; *L2*: ribosomal protein L2; *25S*: 25S rRNA

Subsequently, the geNorm software was employed to determine the stability of the different reference genes (Vandesompele et al. 2002). This program calculates the average expression stability value (M) of each reference gene as the average pair-wise variation (V) between a particular reference gene and all other reference genes (Fig. 2). First, geNorm analysis was performed using all reference genes, which clearly revealed that *ACT11* was less stable than *ACT* (data not shown). This result was confirmed by the Ct values obtained, as *ACT11* (ΔCt = 3.6) showed larger variability than *ACT* (ΔCt = 2.6), and by Normfinder analysis (Table 3). Since it is recommended to include only one reference gene per biological pathway in geNorm analysis (Vandesompele et al. 2002), the data from the *ACT11* reference gene were discarded for further analysis using the geNorm algorithm. For the in vitro leaf samples, all reference genes exhibited an M-value lower than the default threshold of 1.5, indicating that they were suitable for further geNorm analysis. The most stable reference genes were *ACT* and *L2* and the least stable genes were *EF1* and *25S* (Fig. 2a-1). The geNorm algorithm also determines the pair-wise variation (V_*n*_/V_*n*+1_), a measure that is used to determine how many additional reference genes should be included in the calculation of the normalization factor for gene expression. A cut-off V-value of 0.15, below which the inclusion of additional reference genes is not required, has been proposed by Vandesompele et al. (2002). For the in vitro plant samples, the best combination is *ACT* and *L2* (Fig. 2a-1) but it is not adequate with a V-value of 0.19 (Fig. 2b, V2/3), and the addition of one (V3/4) or two (V4/5) reference genes resulted in even higher V-values (Fig. 2B). Thus, no suitable combination of reference genes could be identified for these samples.

### Analysis of candidate reference genes in greenhouse leaves harvested at different developmental stages and from different varieties

RNA was isolated from leaf samples of six Tuu Gia plants at three different time points (Ta, Tb, and Tc) (Fig. 1b). Additionally, for the last time point (Tc), six samples of variety Yangambi Km5 were also harvested (Fig. 1c). The *ACT11* reference gene was not included in these experiments as the experiments described above indicated that *ACT11* is less stable than *ACT* in leaf tissue. Within-group variation was analyzed by ANOVA and showed that the Ct values of all the reference genes except *L2* exhibited statistically significant differences (ANOVA, *p* < 0.05) between leaf samples harvested at different time points, although the differences between the average Ct values of the different groups were smaller than 1.05 Ct except for *ACT* with a difference of 1.4 between time points Ta and Tc. In contrast, the Ct values for *25S*, *L2*, and *TUB* showed significant differences between the two varieties and the Ct differences between the average values for *L*2, *25S*, and *TUB* in the two different varieties is 4.3, 4.1, and 2.0 Ct’s, respectively (Fig. 1c).

For the greenhouse development experiment, NormFinder indicated that *ACT* is the most stable reference gene, and subsequently *25S, EF1*, *TUB*, *L2* and *EF1*, *TUB*, *L2,* *25S* with and without grouping function, respectively (Table 3). NormFinder indicated that the most optimal reference gene combination was *ACT* and *EF1*. For the greenhouse variety experiment NormFinder indicated that *ACT* was the most stable reference gene followed by *EF1*, *TUB*, *25S*, and *L2* and the optimal combination is *25S* and *L2*, which are the two reference genes with more than 4 Ct’s difference between the two varieties*.* GeNorm expression stability analyses of all leaf samples from different time points revealed that all reference genes had an M-value lower than the default threshold of 1.5 (Fig. 2a-2), while for the leaf samples from the two different varieties only *ACT*, *TUB*, and *EF1* had a M-value below 1.5 (data not shown), thus excluding the other reference genes from further analysis. For the greenhouse development experiment, the most stable reference genes were *TUB* and *EF1* and the least stable genes were *25S* and *L2* (Fig. 2a-2). The pair-wise variation analysis indicated that the use of at least three genes (*EF1*, *TUB*, and *ACT*) was optimal as the V-value of 0.15 was obtained (Fig. 2b), which is very similar to the ranking obtained by NormFinder without grouping function. For the variety experiment, no combination resulted in a V-value below 0.15 (Fig. 2B).

### Analysis of reference genes in samples harvested from leaf discs

The Ct variation for samples obtained from the eight leaf discs inoculated with *M. fijiensis* was lower than that of the eight control leaf discs (Fig. 1d) and no statistically significant differences between treatment groups were observed.

NormFinder identified *ACT* as the most stable reference gene and subsequently *TUB* and *L2*, although for *TUB* and *L2* the order interchanged depending on whether the grouping factor was used or not. *25S* and *EF1* were the least stable reference genes (Table 3). The optimal combination according to NormFinder is *ACT* and *TUB*. During geNorm analysis, all reference genes showed an M-value lower than the default threshold of 1.5. The most stable reference genes identified by geNorm were *ACT* and *L2* and the least stable gene was *25S* (Fig. 2A-1). The pair-wise variation analysis indicated that the use of two reference genes (*ACT* and *L2*) was sufficient as the combination yielded a V-value of 0.09 (Fig. 2b). Inclusion of one additional gene (*TUB*) was possible as this combination yielded a V-value of 0.14 (Fig. 2b). For this experiment, geNorm and Normfinder without grouping function yield an identical ranking of the most stable reference genes.

### Analysis of reference genes in shoot meristem cultures

For the sucrose (osmotic stress) experiment, RNA was isolated from five meristems grown either on control medium or on a medium containing a higher sucrose concentration. Additionally, a third group of meristems was harvested 24 h after subculturing to investigate the effect of wounding associated with the cutting and subculturing process. The Ct values of the six different reference genes exhibited the largest variation for *25S* (ΔCt = 2.7) and the least variation for *ACT* (ΔCt = 1.3; Fig. 1e). Differences between the Ct values of the three groups of samples were statistically different for *ACT11* and *TUB* (ANOVA, *p* < 0.05) although the differences between the average Ct values were less than 1 Ct. For the variety experiment, five meristems of Cachaco, Mbwazirume, and Williams grown under standard conditions on the P4 medium were harvested. The Ct value showed the maximum variation for *ACT* (ΔCt = 2.2) and the least variation for *EF1* (ΔCt = 0.7) over all three varieties (Fig. 1f). Statistical differences between the samples of the different varieties were observed for *ACT*, *L2,* *TUB*, and *EF1*, with the largest difference in average Ct values observed between the three varieties of 1.4, 1.2, 0.5, and 0.5, respectively.

For the meristem sucrose experiment, NormFinder analysis showed that *TUB* was the most stable reference gene followed by *L2,* *ACT11*, *EF1*, and *ACT*, although the order interchanged depending on the grouping factor. *25S* was the least stable reference gene (Table 3). For the meristem varieties experiment, *EF1* was the most stable reference gene and subsequently *25S,* *TUB*, *L2*, and *ACT11*, although the order interchanged depending on the Normfinder grouping factor here as well. *ACT* was the least stable reference gene (Table 3). NormFinder indicated *ACT*/*TUB* and *ACT11*/*L2* as the optimal reference gene combinations for the sucrose and varieties experiments, respectively. From Fig. 1e, f and the Normfinder analyses (Table 3), it is clear that *ACT* exhibited as much or more variation than *ACT11*. Therefore, for meristem samples, *ACT* was not included in the geNorm analysis. Using all samples from the sucrose and varieties experiments, geNorm analysis resulted in M-values below the default threshold 1.5 for all reference genes and the most stable reference genes were *ACT11*/*EF1* and *ACT11*/*TUB*, respectively (Fig. 2a-3, -4, respectively). Finally, the pair-wise variation analysis indicated that the use of two genes was sufficient as a V-value of 0.14 (*ACT11* and *EF1*) and 0.09 (*ACT11* and *TUB*) was obtained for the samples of the sucrose and varieties experiments, respectively (Fig. 2b). These genes were also among the three most stable reference genes as identified by the Normfinder analysis without grouping function in each experiment.

## Discussion

Quantitative RT-PCR is one of the most commonly applied methods for the analysis of mRNA expression levels, because of its accuracy and sensitivity. Recent studies have clearly advocated the use of multiple suitable reference genes for normalization of sample gene expression (Bustin 2002; Gutierrez et al. 2008b; Huggett et al. 2005; Vandesompele et al. 2002) and have recommended a thorough assessment of these reference genes for expression stability. Screening multiple reference genes allows distinction between variations in the amount of cDNA input and variations in gene expression. Suitable reference genes for normalization are often selected using software programs such as geNorm (Vandesompele et al. 2002) and NormFinder (Andersen et al. 2004). Recent studies have indicated that the traditional reference genes are not always stably expressed in different species, tissues, and experimental treatments (Artico et al. 2010). For example, in *Arabidopsis* the reference genes coding for actin, tubulin, ubiquitin, and elongation factor showed high variability (Gutierrez et al. 2008a), confirming the need for the assessment of even traditional reference genes in a specific species and under relevant environmental treatments. A recent study in banana by Chen et al. (2011) also strongly suggests that a thorough validation of the stability of candidate reference genes under specific experimental conditions is required.

*Musa* is a non-model plant with limited sequence information available, and thus a limited number of candidate reference genes. Therefore, in this study, we selected candidate reference genes for which such sequence information was publicly available. RT-qPCR protocols were developed for four different reference genes (*ACT11*, *EF1*, *L2*, and *TUB*) as well as two previously reported reference genes in *Musa* (*ACT* and 25S RNA). Recently, Chen et al. (2011) selected 18 candidate reference genes from a proprietary banana transcriptome sequence database. The stability of expression of these genes and that of two additional genes from publicly available sequences was analyzed in six sample sets, all originating from the Cavendish dessert banana. None of these genes was researched in the present investigation although members of the same gene families were analyzed in both studies (actin, elongation factor 1α, ribosomal protein L, and tubulin). Due to a non-specific amplification we did not process the ubiquitin gene, while Chen et al. (2011) validated the usefulness of the *UBQ2* gene despite a similar problem of non-specific amplification.

Factors known to affect the reliability of gene expression data such as RNA quality, DNase I treatment, two-step RT-qPCR, PCR efficiency, and non-specific amplification were controlled (Derveaux et al. 2010; Maroufi et al. 2010). Of the two reference genes that were annotated as actin genes, only the gene exhibiting the lowest level of Ct variation in the samples was retained for further analysis with the geNorm algorithm. Hence, for in vitro leaf samples, *ACT11* was excluded from the geNorm analyses, whereas for meristem samples these analyses were executed without *ACT*. Similarly, in fruit tissues of dessert banana, differences were seen in the expression stability of the different actin genes analyzed (Chen et al. 2011).

Our study showed that the expression levels of all reference genes investigated exhibited high Ct variability in the leaf samples of the *Musa* plants grown in vitro. No significant Ct differences between the control group and the acetone-treated group were identified. NormFinder identified *L2* as the most stable reference gene and subsequently *TUB*. Furthermore, the software analysis revealed that the combination of these two reference genes would give the most reliable gene expression outcome. The geNorm algorithm indicated that the combination of the *ACT* and *L2* reference genes is preferred although it is not sufficient to normalize gene expression levels in these in vitro leaf samples. Moreover, the use of additional reference gene(s) resulted in even more unacceptable reference gene combinations for normalization, indicating that suitable reference genes for in vitro gene expression studies are scarce. These results also suggest that plants grown in vitro might be stressed and show variable expression levels of genes involved in basic biological processes. Analysis of the expression of genes of interest in such samples is thus difficult and requires careful examination of candidate reference genes prior to any analysis.

For greenhouse leaf samples harvested at different developmental stages and for leaf discs, the geNorm analysis demonstrated that the combinations *EF1*/*TUB*/*ACT* and *L2/ACT*, respectively, allow reliable normalization despite the occurrence of significant Ct differences between different sample groups in the former for all but one (*L2*) reference gene. NormFinder analyses resulted in similar results and indicated that the combinations *ACT/EF1* and *ACT*/*TUB* are optimal for normalization of leaf samples at different developmental stages and leaf discs, respectively. For leaf samples from different varieties the ANOVA indicated significant differences for *L2*, *25S*, and *TUB* with large differences (ΔCt > 4.0) in average Ct’s for *L2* and *25S*, which were both excluded from the geNorm analysis, resulting in the inability to identify a suitable combination of reference genes. NormFinder identified *ACT* as most stable and *L2* and *25S* as least stable reference genes, but surprisingly indicated *L2* and *25S* as the most suitable reference gene pair. A glance at the raw Ct’s shows that these genes are relatively stable within each variety, but the level of *L2* and *25S* is ±4 Ct’s higher and ±4 Ct’s lower, respectively, in Km5 than in TG (Fig. 1). The recent reference gene validation study in Cavendish banana by Chen et al. (2011) mostly dealt with fruit tissues (141 out of 144 samples). The only tissue common to this study and our study is leaf tissue, but it was isolated from mature plants in the field whereas we sampled in vitro and greenhouse plants. For the sample set examining different tissues including three leaf samples, *ACT2* was the third most stable gene and the preferential pair included a GTP-binding nuclear protein encoding gene and a ribosomal protein 2 gene (Chen et al. 2011).

In meristem cultures a different set of reference genes seemed more stable than in leaf samples, although some statistically significant Ct differences between the sample groups were observed in these tissue samples as well. The geNorm algorithm yielded multiple reference gene combinations useful for both the sucrose and varieties experiments. The minimum suitable combinations for gene expression normalization are *ACT11*/*EF1* and *ACT11*/*TUB*, respectively. Alternatively, NormFinder proposed *TUB*/*ACT* and *L2*/*ACT11* as the most suitable reference gene combinations for the sucrose and varieties experiments, respectively.

Numerous reference gene expression studies have used both geNorm and NormFinder (Barsalobres-Cavallari et al. 2009; Chen et al. 2011; Cruz et al. 2009; Exposito-Rodriguez et al. 2008; Hong et al. 2008; Hu et al. 2009; Lee et al. 2010; Maroufi et al. 2010; Paolacci et al. 2009; Remans et al. 2008) and reported limited variation in stability ranking by these software tools, whereas other studies have reported significantly different results depending on the sofware (Lin and Lai 2010; Paolacci et al. 2009; Schmidt and Delaney 2010). These variations stem from differences between geNorm and NormFinder in the mathematical approaches used to calculate expression stability. We have noted that without grouping function, the rankings by NormFinder and geNorm were more consistent than when using the grouping function of Normfinder. GeNorm determines the stability of the candidate reference gene against that of all other candidate reference genes under investigation by pair-wise comparison of variation of expression ratios. One of the drawbacks of geNorm is that it is sensitive to co-regulation (Vandesompele et al. 2002), which is why it is important to use reference genes involved in different biological processes. Further, geNorm identifies the appropriate number of reference genes for accurate normalization, whereas NormFinder selects two genes with minimal combined inter- and intra-group expression variation to take into account systematic differences between sample subgroups. Our results confirm the observation of Rytkönen et al. (2010) that ANOVA tests in some cases indicate statistically significant differences between sample groups for reference genes that were ranked by NormFinder and/or geNorm as the most stable candidate genes. From the greenhouse leaf variety experiment and its Normfinder analysis it becomes clear that the use of reference genes showing significant different expression levels in different varieties might still be considered suitable when used in combination. However, it should be noted that geNorm analysis of these samples failed to identify a suitable reference gene combination.

The expression levels of some of the reference genes investigated clearly differed between banana varieties tested. Nevertheless, for most of these genes the level of stability seemed similar across different varieties. This suggests that reference genes validated in one banana variety might be suitable candidates in other banana varieties, but this should always be confirmed prior to expression studies of genes of interest. Based on our results, we propose the use of *ACT*, *TUB*, and *EF1* for reliable normalization of gene expression in banana leaf samples and multiple combinations of *TUB*, *ACT*, *ACT11*, *EF1*, and *L2* for gene expression studies in banana meristem cultures. Similarly, Chen et al. (2011) concluded that each experimental condition tested demands a specific set of reference genes, since for the six banana sample sets analyzed six different pairs of optimal reference genes were identified. Graeber et al. (2011) concluded that for different Brassicaceae species the reference gene expression stability is higher for a given developmental process between distinct species than for distinct developmental processes within a given single species.

Of the candidate reference genes evaluated in this study, *ACT* and *25S* have been used previously as single “controls” in banana (Mbeguie-Mbeguie et al. 2007; van den Berg et al. 2007). More specifically, the reference gene *ACT* was used as the control gene in an experiment investigating changes in gene expression during fruit development (Mbeguie-Mbeguie et al. 2007). In another study involving *Fusarium* wilt-infected roots, the *25S* gene was used for normalization of gene expression (van den Berg et al. 2007). Neither of these studies reported on the stability of the reference gene under the experimental conditions investigated. This information is also lacking in the expression studies of the newly discovered banana dehydrin gene (Shekhawat et al. 2011) and MADS-box genes (Elitzur et al. 2010), although two reference genes were included in these reports (*ACT*/*EF1α* and a ribosomal RNA gene/*GAPDH*, respectively). In our study, *ACT* was found to be one of the most stable reference genes whereas Chen et al. (2011) revealed that the selected banana actin genes were not within the preferential pair for five of the six experimental conditions. The results presented here also showed that the *25S* gene was relatively unstable in leaf tissues. This study confirms that multiple reference genes should be screened for each tissue type and stress condition. The identification of reliable reference genes is time-consuming and expensive but at the same time necessary for accurate gene expression analyses. The present study provides a strong set of candidate reference genes for researchers working on *Musa* gene expression in leaf and meristem tissues from different banana varieties and complements the study of Chen et al. (2011) that mainly deals with fruit tissues.

In summary, this is a detailed study aimed at validating candidate reference genes for the quantification of transcript levels in various banana varieties under different experimental conditions and in different non-fruit tissues. Identification of suitable reference genes for normalization is indeed challenging in the case of some tissues and conditions. In our study, this was the case for in vitro leaf samples. We recommend classical reference genes, namely *EF1*, *ACT*, and *TUB*, and appropriate primer sequences as references for normalization in expression studies in leaves of greenhouse plants, *ACT* and *L2* for leaf discs, and we advocate the use of combinations of *TUB*, *ACT*/*ACT11*, and *EF1* for expression studies in meristems.

## Electronic supplementary material

Below is the link to the electronic supplementary material.

Supplementary material 1 (DOC 33 kb)
