# Supplementary material for: Selection and validation of reference genes for quantitative RT-PCR expression studies of the non-model crop Musa
Source: Mol Breed. 2012 Jun 8;30(3):1237–52. doi: 10.1007/s11032-012-9711-1 (PMC3460175; doi:10.1007/s11032-012-9711-1)
Supplement: Supplementary file 1 — Supplementary material 1 (DOC 33 kb) [file 11032_2012_9711_MOESM1_ESM.doc]

**Electronic Supplementary Table** 1. Description of banana candidate reference genes and comparison with orthologs

| Gene name (abbreviation) | *Arabidopsis* or rice accession number | *Musa* accession number identified by BlastX (% identity) | No. of PCR products^a^ |
| --- | --- | --- | --- |
| Actin11 (*ACT*11) | At3G12110 | AF285176 (83) | 1 |
| Actin (*ACT*) | ND | Mbeguie—Mbeguie et al. 2007 ^b^ | 1 |
| Cyclophilin | At4G03870 | S_600130742T1 (97)^c^ | 0 |
| Elongationfactor-1α (*EF*1) | At5G60390 | C_600124753T1 (85)^c^ | 1 |
| Ribosomal protein L2 (*L2*) | Os12G01567700 | C_600158347T1 (88)^c^ | 1 |
| 25S rRNA (*25S*) | ND | van den Berg et al, 2007^b^ | 1 |
| β-tubulin (*TUB*) | Os06g0671900 | C_600133138T1 (86)^c^ | 1 |
| Ubiquitin | At4G05320 | AF502575 (80) | 2 |

^a^As determined by gradient PCR.

^b^Previously published reference genes from *Musa* species.

^c^Accession number from Musa 3′ EST database donated to the Global Musa Genomics Consortium by Syngenta.

Abbreviation: ND, not determined.
